# Cancer-associated fibroblasts-derived lncRNA signature as a putative biomarker in breast cancer

**DOI:** 10.3389/fonc.2022.1028664

**Published:** 2022-11-02

**Authors:** Zan Li, Junyi Yu, Chunliu Lv, Zhenhua Luo

**Affiliations:** Department of Oncology Plastic Surgery, Hunan Cancer Hospital, The Affiliated Cancer Hospital of Xiangya Medical School, Central South University, Changsha, Hunan, China

**Keywords:** immunotherapy, lncRNA, prognosis, cancer-associated fibroblasts, breast cancer

## Abstract

Long noncoding RNAs (lncRNAs) have been reported to play a key role in regulating tumor microenvironment and immunity. Cancer-associated fibroblasts (CAFs) are abundant in many tumors. However, the functional and clinical significance of lncRNAs specifically expressed in CAFs has not been fully elucidated. In this study, we identified a list of 95 CAF-specific lncRNAs (FibLnc), including *HHLA3*, *TP53TG1*, *ST7-AS1*, *LINC00536*, *ZNF503-AS1*, *MIR22HG*, and *MAPT-AS1*, based on immune cell transcriptome expression profiling data. Based on the Cancer Genome Atlas and Gene Expression Omnibus datasets, we found that the FibLnc score predicted differences in overall patient survival and performed well in multiple datasets. FibLnc score was associated with the clinical stage of patients with breast cancer but did not significantly correlate with the PAM50 classification. Functional analysis showed that FibLnc was positively correlated with signaling pathways associated with malignant tumor progression. In addition, FibLnc was positively correlated with tumor mutational load and could predict immunotherapy response in patients with breast cancer receiving anti-PD-1 or anti-CTLA4 therapy. Our proposed FibLnc score was able to reflect the status of the immune environment and immunotherapeutic response in breast cancer, which could help explore potential therapeutic decisions and regulatory mechanisms of CAF-specific lncRNAs.

## Introduction

Breast cancer is one of the most common cancers worldwide and is the second leading cause of tumor-related deaths in women (1–3). The treatment options for breast cancer usually include a combination of surgical excision, radiation therapy, and drug therapy (hormonal therapy, chemotherapy, and/or targeted biologic therapy) to treat microscopic cancer that spreads from the breast tumor through the bloodstream (4). Breast cancer patients have a good prognosis in early diagnosis, but only around 25% will survive their cancer for 5 years or more after they are diagnosed at stage IV (5). Therefore, there is an urgent need to develop new molecular targets and therapeutic strategies.

The tumor microenvironment (TME), which has been a topic of interest, contains stromal cells, immune cells, and noncellular components that may influence the diagnosis and prognosis of patients with breast cancer (6, 7). Various tumors actively engage with their microenvironment, which is a factor that strongly influences tumor progression and metastasis. TME has been shown to be an important cause of tumor resistance to antichemotherapy drugs (8, 9). In addition, some immune cells, such as macrophages, secrete TGF-β, which reduces the abundance of succinate dehydrogenase, and promotes increased glycolysis, thus enhancing tumor growth and immunosuppression (10, 11).

Recently, numerous studies have shown that breast cancer-associated fibroblasts play a role in the development and progression of breast cancer, and cancer-associated fibroblasts (CAFs) are the most abundant cellular component of the breast cancer microenvironment, with high expression of many growth factors, such as hepatocyte growth factor, transforming growth factor beta, and fibroblast growth factor. Most of these genes promote invasion and metastasis (12, 13). Breast cancer-associated fibroblasts can also regulate triamcinolone resistance through activation of the MAPK and PI3K/Akt pathways and phosphorylation of ERα (14). These findings suggest that studies on CAFs in the breast cancer microenvironment may further elucidate the complex relationship between cancer cells and their microenvironment and identify new targets for the treatment of breast cancer. Long noncoding RNAs (lncRNAs) have been reported to play a key role in regulating TME and tumor immunity (15). However, the functional and clinical significance of lncRNAs specifically expressed in CAFs have not been fully elucidated. Therefore, there is an urgent need to explore the potential therapeutic decisions and regulatory mechanisms of CAF-specific lncRNAs.

In this study, we generated a CAF-specific lncRNA (FibLnc) score that could predict the differences in overall patient survival and perform well in multiple datasets. The FibLnc score was found to be associated with the clinical stage of patients with breast cancer and signaling pathways related to malignant tumor progression. In addition, FibLnc was positively correlated with tumor mutational load and could predict immunotherapy response in patients with breast cancer receiving anti-PD-1 or anti-CTLA4 therapy. The robust and powerful FibLnc score was able to reflect the immunotherapeutic response in breast cancer and provide insightful suggestions for exploring potential therapeutic decisions and regulatory mechanisms of CAF-specific lncRNAs.

## Results

### CAFs are the major components in breast cancer TME

**Figure 1:**
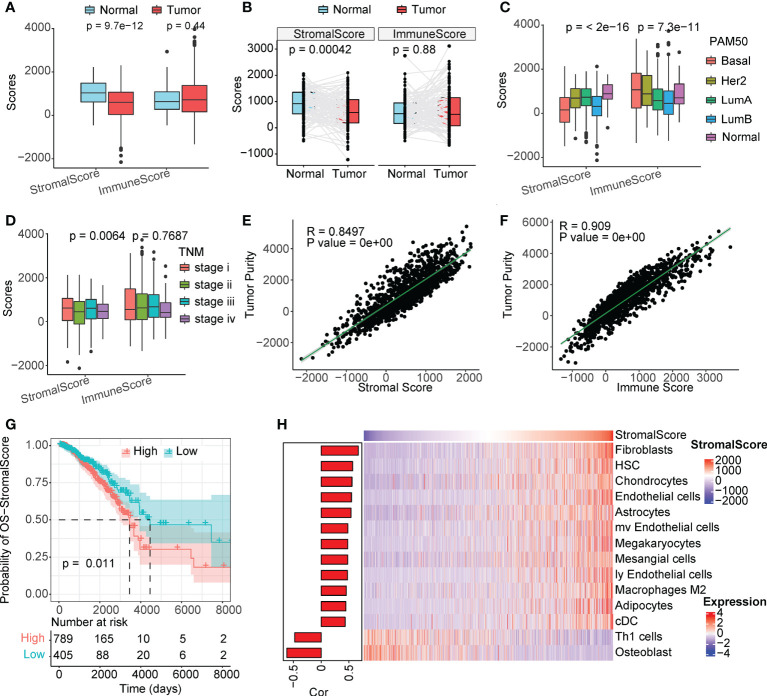
Stromal scores are associated with clinical features and outcomes. **(A)** Analysis of unpaired differences in the distribution of immune and stromal scores in tumors and normal tissues adjacent to the tumor. **(B)** Analysis of pairwise differences in immune and stromal score distribution in tumors and normal tissues adjacent to the tumor. **(C)** Analysis of the differences in the distribution of immune and stromal scores among different PAM50 subtypes. **(D)** Analysis of the differences in the distribution of immune and stromal scores among different TNM stages. **(E, F)** Association between immune/stromal scores and tumor purity inferred using the ESTIMATE algorithm. **(G)** Survival analysis of the low and high stromal scores. **(H)** Correlation between TME cell abundance and immune score.

The overall immune and stromal infiltration levels and tumor purity were calculated for The Cancer Genome Atlas (TCGA) breast cancer samples (**Table S1**). We analyzed the differences in immune and stromal infiltration scores between the tumor and normal patients. Expectedly, the stromal infiltration score was significantly higher in normal samples than in tumor samples, and a similar phenomenon was observed in paired samples (**Figures 1A, B**). However, the level of immune infiltration did not differ significantly between the tumor and normal patients. When further comparing the correlation of clinical factors with cellular infiltration scores in patients with breast cancer, its association with stromal infiltration level was more significant than with immune infiltration scores (**Figures 1C, D**, and **S1**). The stromal infiltration level was more significantly different in different PAM50 subtypes than immune infiltration level, interestingly, we also found that immune infiltration level was not significantly associated with tumor stage and age of patients, but stromal infiltration level were significantly different in different groups of samples. These suggesting that stromal infiltration level may be more associated with tumor progression. Furthermore, the immune and stromal scores were significantly correlated with tumor purity (**Figures 1E, F**). In addition, we found that the level of stromal infiltration was significantly negatively associated with survival (**Figure 1G**), while that of immune infiltration was associated with a better prognosis in patients with breast cancer (Figure S1F). As shown in **Figure 1H**, CAFs were the most relevant cells in the microenvironment of patients with breast cancer with the level of stromal infiltration, suggesting a potential crucial function of CAFs in regulating breast cancer tumor microenvironment. Previous studies have shown that CAFs suppress the activity of immune cells, leaving tumor patients in an immunosuppressed state (16, 17), which is consistent with the opposite prognostic predictive value of immune and stromal infiltration scores in patients with breast cancer.

### Construction of FibLnc risk score model

**Figure 2:**
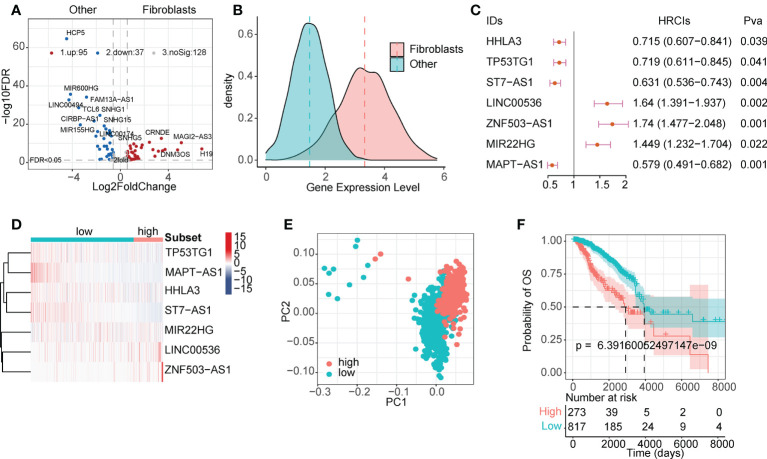
CAF-specific lncRNAs identification and model construction. **(A)** Differentially expressed lncRNAs between CAF and other TME cells. **(B)** The density plot of expression values of CAF-specific lncRNAs and other lncRNAs. **(C)** Forest plot of the seven lncRNAs used for model construction. **(D)** Heatmap of the expression level of eight lncRNAs used for model construction. **(E)** PCA analysis of the high- and low-risk subgroups. **(F)** Kaplan–Meier survival curves for patients in TCGA database assigned to high- and low-risk groups based on the risk score.

LncRNAs play a regulatory role in the TME. Based on single-cell line expression matrix data from several databases, 95 lncRNAs were identified to be specifically highly expressed in CAFs (**Figure 2A** and **Table S2**). As shown in **Figure 2B**, the expression of these lncRNAs was significantly higher in CAFs than in normal cells. Next, we screened survival-related lncRNAs in breast cancer using univariate Cox regression analysis (**Table S3**) and constructed LASSO-Cox risk regression models based on these lncRNAs. After screening (**Figure S2**), the final seven lncRNAs, namely *HHLA3*, *TP53TG1*, *ST7-AS1*, *LINC00536*, *ZNF503-AS1*, *MIR22HG*, and *MAPT-AS1*, were used to calculate the FibLnc score. These lncRNAs were associated with the overall survival of patients with breast cancer and were differentially expressed in the high- and low-risk groups (**Figures 2C, D**). Principal component analysis showed significant differences in gene expression patterns between high- and low-risk groups (**Figure 2E**), and survival analysis further suggested that FibLnc scores were associated with worse overall patient survival (**Figure 2F**). We further validated this in several datasets (**Figure S3**) and found that the FibLnc score significantly differentiated patient overall survival.

### Evaluation of FibLnc risk score model

**Figure 3:**
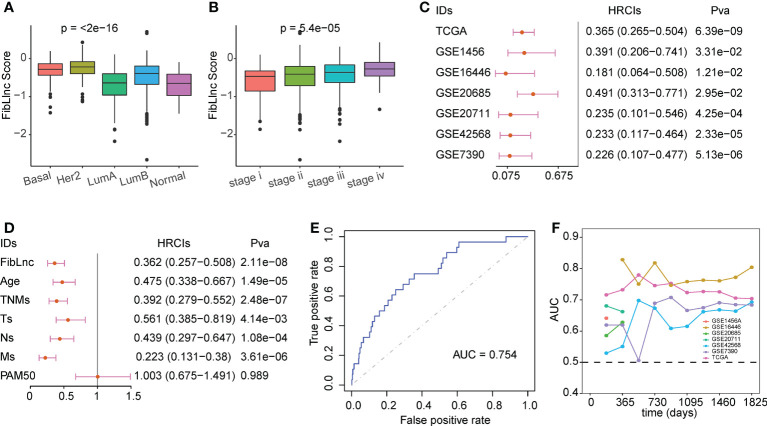
Evaluation for the prognostic value of the FibLnc score. **(A)** Analysis of the differences in the distribution of FibLnc scores among different PAM50 subtypes. **(B)** Analysis of the differences in the distribution of FibLnc scores among different TNM stages. **(C)** Forest plot of Cox analysis in TCGA and Gene Expression Omnibus (GEO) datasets. **(D)** Forest plot of Cox analysis of the FibLnc score and clinical features. **(E)** ROC curve of FibLnc scores used for survival status prediction. **(F)** Time-dependent area-under-the-curve value in TCGA, GSE1456, GSE7390, GSE16446, GSE20685, GSE20711, and GSE42568.

We compared the association between FibLnc score and different clinical factors and found that FibLnc score was significantly associated with age, PAM50 subtypes, and tumor node metastasis stages (**Figures 3A, B**; **S4A**). As shown in the figure, Basal and HER2+ subtypes exhibited highest FibLnc score, which is consistent with that patients classified into these subtypes have worse prognosis. The similar phenomenon could be seen in the association of tumor stage and FibLnc score. In contrast, a weak correlation was observed between the FibLnc score and the level of immune and stromal infiltration (Figure S4B). The FibLnc score showed good prognostic predictive power in several breast cancer gene expression datasets (**Figure 3C**). We further compared the prognostic assessment ability of the FibLnc score compared to clinical characteristics, and the results showed that the FibLnc score exhibited comparable or even slightly stronger performance (**Figure 3D**). In addition, the AUC of the FibLnc score in predicting patient survival status was 0.754 (**Figure 3E**) and showed a considerable performance in predicting survival beyond three years in different datasets (**Figure 3F**). We further constructed a nomogram to demonstrate the predictive performance of FibLnc scores compared to clinical factors. As shown in **Figure S5**, the FibLnc score showed stable and excellent performance in predicting one-, three-, five-year survival.

### Functional analysis of FibLnc risk score

**Figure 4:**
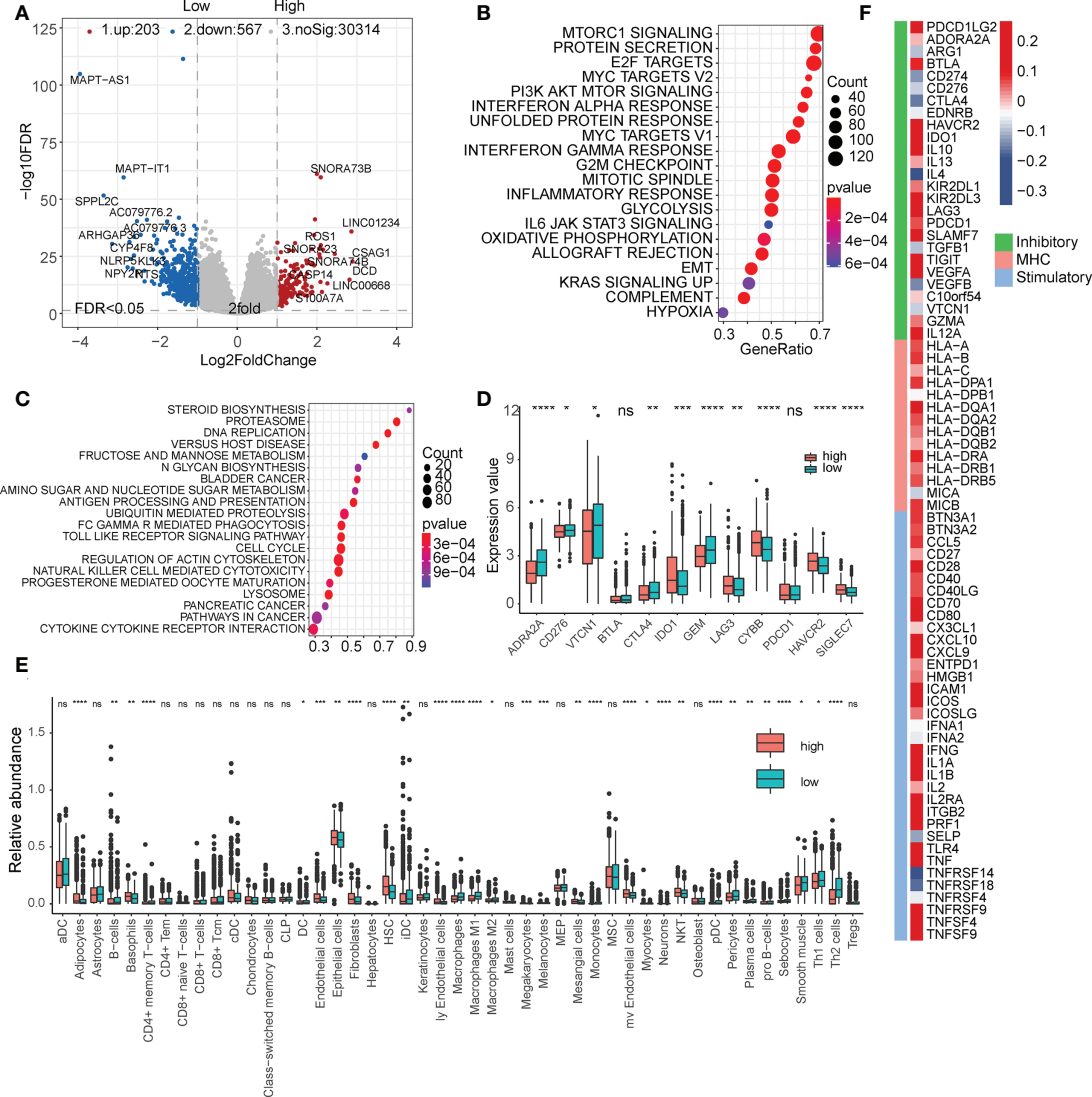
Functional analysis of the FibLnc score and breast cancer immune signature. **(A)** Differentially expressed genes between high- and low-risk subgroups. **(B)** Hallmark enrichment analysis of the distribution of FibLnc scores. **(C)** KEGG enrichment analysis of the distribution of FibLnc scores. **(D)** Analysis of the differences in the distribution of immune checkpoints between high- and low-risk subgroups. ns means P > 0.05, * means P ≤ 0.05, ** means P ≤ 0.01, *** means P ≤ 0.001, **** means P ≤ 0.0001. **(E)** Analysis of the differences in the distribution of immune cells between high- and low-risk subgroups. ns means P > 0.05, * means P ≤ 0.05, ** means P ≤ 0.01, *** means P ≤ 0.001, **** means P ≤ 0.0001.

We then analyzed the differentially expressed genes in the high- and low-risk subgroups (**Figure 4A** and **Table S4**). The high-risk subgroup significantly overexpressed LINC01234, which is known to be a key marker for tumor proliferation and metastasis. The enrichment analysis of hallmark and KEGG pathways suggested that many oncogenic pathways, such as mTOR signaling, MYC targets, E2F targets, and DNA replication signaling pathways, were significantly activated in high-risk groups (**Figures 4B, C**, **Table S5**, and **Table S6**). In addition, we also found that some immune checkpoints, such as CTLA4, ADRA2A, and GEM, were significantly upregulated in the low-risk group (**Figure 4D**). These results suggest that our FibLnc scores may be associated with the immune microenvironment. Based on the relative abundance of immune and stromal cells calculated using xCell, we found that the proportion of tumor-associated stromal cells was significantly higher in the low-risk group (**Figure 4E**). We further analyzed the relationship of FibLnc scores with immune-related regulators and found that it was significantly negatively correlated with the expression of some immunosuppressive factors, such as CTLA4 and PD1, and positively correlated with the expression of most MHC family members and immune-stimulating factors (**Figure 4F**).

### FibLnc risk score is associated with mutation status and drug response

**Figure 5:**
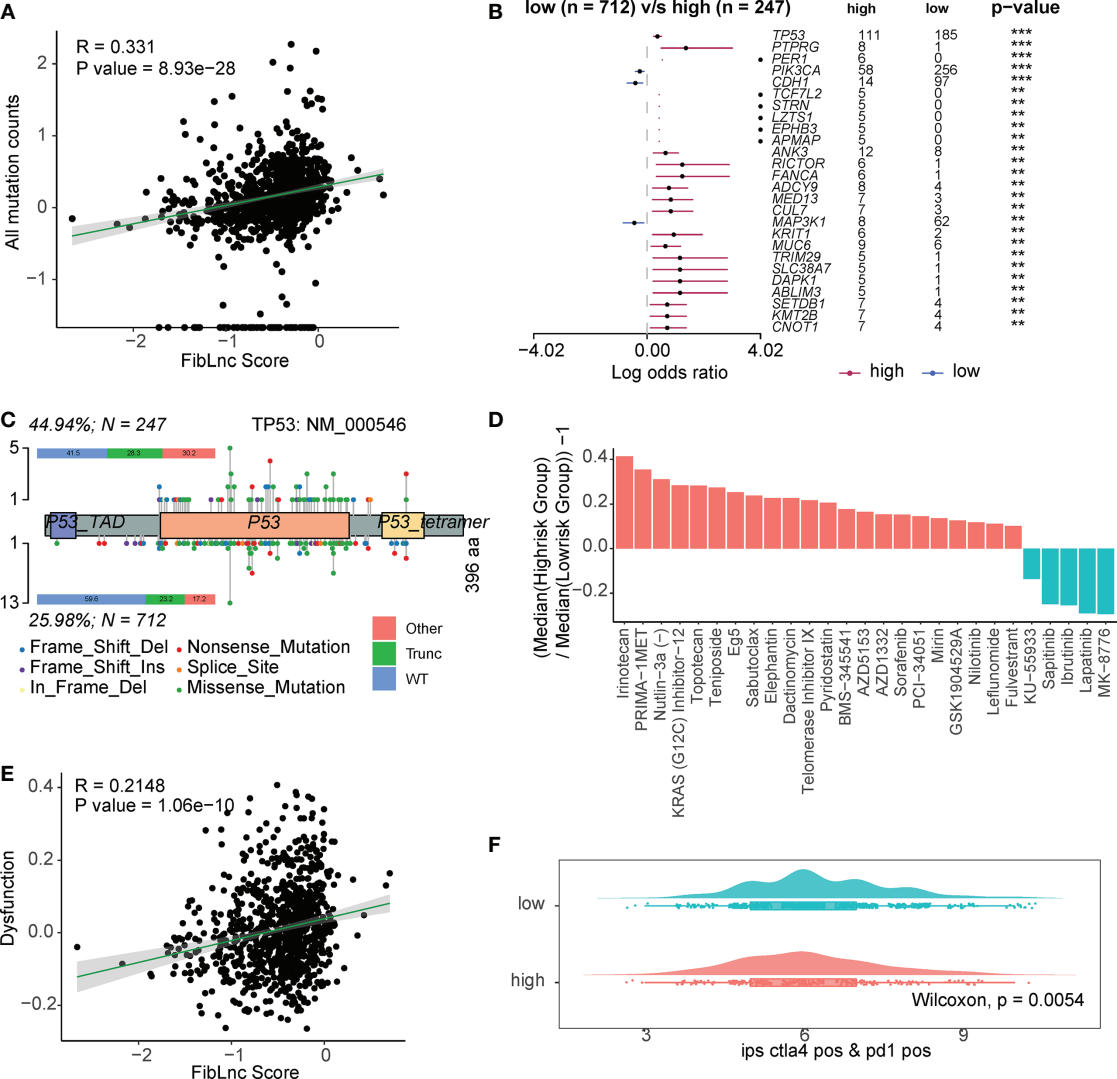
The FibLnc score predicts therapeutic benefits. **(A)** Correlation analysis of all mutation counts and FibLnc scores. **(B)** Mutation landscape difference between high- and low-risk subgroups. **(C)** Lollipop chart displaying mutation sites of TP53 proteins. **(D)** The ratio of normalized IC50 value of the 198 drugs between the high- and low-risk subgroups. **(E)** Correlation analysis of tumor-infiltrating immune cell dysfunction scores and FibLnc scores. **(F)** Distribution of IPS score of patients under anti-CTLA-4 or anti-PD-1 treatment between high- and low-risk subgroups.

We further analyzed the relationship between FibLnc score, mutation status, and drug response. As shown in **Figure 5A**, FibLnc scores and mutation counts were significantly positively correlated. Many oncogenes, such as *TP53* and *PIK3CA* (**Figure 5B** and **Figure S6**), were mutated more frequently in the high-risk group and may be associated with a worse prognosis in the group. We also found more truncation-related mutations in *TP53* in the high-risk group than in the low-risk group (**Figure 5C**). In addition, the patterns of co-occurrence and mutually exclusive mutations in the high- and low-risk subgroups were also very different; for example, *TP53* and *PIK3CA* were more mutually exclusive in the low-risk group, but this phenomenon was not observed in the high-risk group (**Figure S7**). We further predicted the response of the high- and low-risk groups to the drugs (**Table S7**) and found that the high-risk subgroup responded significantly to irinotecan, PRIMA-1MET, topotecan, etc., while the low-risk group responded to MK-8776, lapatinib, ibrutinib, etc (**Figure 5D**). We also found a significant positive correlation between FibLnc score and immune cell dysregulation score (**Figure 5E**) and a significantly stronger immune response to CTLA4 and PD1 inhibitors in the low-risk group than in the high-risk group (**Figures 5F** and **S8**).

## Discussion

The TME consists of a series of stromal cells, immune cells, and noncellular components that may influence the diagnosis and prognosis of patients with breast cancer (6, 7). In this study, we evaluated the level of stromal and immune infiltration in TCGA breast cancer samples according to the ESTIMATE algorithm and found that the level of stromal infiltration was substantially lower in tumors than in normal samples, whereas that of immune infiltration was not significantly different, and the stromal score was significantly associated with patient survival. Based on this, we found that the abundance of CAFs was most associated with the level of stromal infiltration in patients with breast cancer. It has been shown that CAFs are a subpopulation of fibroblasts that promote tumor progression and metastasis by secreting various chemokines, cytokines, and degrading extracellular matrix proteins (18). CAFs can suppress Th1 immune responses by inhibiting Th1 cytokines while enhancing the immunosuppression of Th2 cytokines to promote tumor growth (19). Breast cancer-associated fibroblasts significantly enhance the invasive and migratory ability of the T47D breast cancer cell line (20, 21).

In recent years, it has been shown that the expression of some lncRNAs is cell-specific and their expression patterns are closely related to the tumor immune microenvironment. There are several studies of cancer-associated fibroblasts-derived lncRNAs affecting tumor cell signaling pathway changes previously. Zhang et al. (22) reported DNM3OS, a CAF-promoted lncRNA, influenced radio-resistance in esophageal squamous cell carcinoma through controlling the DNA damage response. Our study was a comprehensive analysis of CAF-related lncRNAs, whereas their study concentrated on the biological function of a particular CAF-related lncRNA. Using machine learning techniques, Liu et al. (23) created an immune-derived lncRNA profile for enhancing colorectal cancer outcomes. Instead of concentrating solely on CAF-related lncRNAs, this study also included immune-derived lncRNAs. LncRNA signatures have been widely described in colorectal cancer and are strongly associated to a number of biological activities, including cell death (24), epigenetic alteration (25), and tumor immunity (23). To better understand the mechanisms of CAF- derived lncRNAs in breast cancer, we identified CAF-specific lncRNAs called FibLnc and constructed a survival risk assessment model for patients with breast cancer. The FibLnc score has considerable potential for predicting patient survival status. Cross-validation showed that the FibLnc score performed well in various breast cancer gene expression datasets and showed high robustness in predicting survival probability. We performed a comprehensive review of the mechanisms and prognostic values of the seven lncRNAs used for modelling, all of them are associated with cancer progression, especially for *MAPT-AS1*, which is the most relevant gene with FibLnc score, is proved to be correlated with the cell growth, invasiveness and paclitaxel resistance in breast cancer cells through antisense pairing with *MAPT*. *MAPT-AS1* may serve as a potential therapeutic target in ER-negative breast cancers (26).

Differential expression analysis showed that *LINC01234* was the most differentially expressed gene. Several studies have shown that *LINC01234* is closely associated with tumor cell proliferation and metastasis (27–29). *LINC01234* has emerged as an important regulator that is upregulated in colon cancer and is associated with poor prognosis (30), and its knockdown significantly inhibitted tumorigenesis in hepatocellular carcinoma (31). In addition, mutational analysis showed that the high-risk group contained more mutations in cancer-related genes, such as *TP53* and *PIK3CA*, which have been previously reported to be closely associated with cancer development (32, 33). This may explain why patients in the high-risk group had a worse prognosis.

In the drug sensitivity analysis, we found that the FibLnc score can help predict potential target agents. Some drugs, such as irinotecan, PRIMA-1MET, topotecan, MK-8776, lapatinib, and ibrutinib, showed different responses between the subgroups. In assessing patient response to anti-PD-1 or anti-CTLA4 immunotherapy, patients in the low-risk subgroup had relatively higher immunophenoscore (IPS), significantly lower immune cell dysfunction scores, and high expression of immune checkpoints, such as CTLA4 and PD-1, suggesting that they may respond better to immunotherapy.

However, limitations still exist. the signature was built and validated using retrospective samples, validation using prospective real-world samples was also required. Furthermore, our conclusions are mainly obtained by bioinformatics analysis and lack critical experimental validation. Although we performed cross-validation on multiple datasets to evaluate the robustness of the model, immunohistochemical validation of the expression of these modeled genes was necessary. Finally, we expounded the function and clinical significance of CAF-associated lncRNAs in breast cancer, but the molecular mechanism is still lacking. We need to carry out exhaustive verification of our analysis results in the future to clarify the biological mechanisms of CAF-associated lncRNAs in breast cancer.

## Conclusions

In conclusion, we generated a FibLnc score that could predict the differences in overall patient survival and was found to perform well in multiple datasets. The robust and powerful FibLnc score was able to reflect the immunotherapeutic response in breast cancer and provide insightful suggestions for exploring potential therapeutic decisions and regulatory mechanisms of CAF-specific lncRNAs.

## Methods

### Gene expression dataset preparation

The level 3 gene expression matrix (log2 normalized) of breast cancer samples and corresponding clinical information from The Cancer Genome Atlas (TCGA) were downloaded from Xena Browser (https://xenabrowser.net/datapages/). The microarray matrices GSE1456, GSE7390, GSE16446, GSE20685, GSE20711, and GSE42568 and their corresponding clinical information were downloaded from the Gene Expression Omnibus database (https://www.ncbi.nlm.nih.gov/geo/). Gene expression matrices were collected from several cohorts, including the Human Primary Cell Atlas (34), Encyclopedia of DNA Elements (35), Blueprint (36), Database of Immune Cell Expression (37), and GSE107011. All matrices were then combined and normalized using the ComBat function of the R package ‘sva’ v3.42.0 (38) for further analysis.

### Immune cell abundance and score estimation

xCell (39) was used to evaluate the relative abundance of immune and stromal cells in breast cancer samples based on the log2-transformed gene expression matrix downloaded from TCGA. The overall immune and stromal infiltration levels and tumor purity were calculated using the R package ‘estimate’ v1.0.13 (40).. First, we prepared the gene expression matrix of TCGA, then converted it into GCT format, and filter the genes of the matrix with the gene signature related to immune and stromal infiltration, and finally converted the gene expression matrix to immune and stromal infiltration matrix based on the ESTIMATE algorithm. The detailed process can be found in R package ‘estimate’ v1.0.13 and our source code (https://github.com/kodayu/FibLnc.git). The default parameters were used.

### Identification of differentially expressed genes

Based on the downloaded count matrix of immune cell gene expression, we performed differential expression analysis using the R package ‘DESeq2’ (v1.30.1) to compare lncRNAs aberrantly expressed between fibroblasts and immune or stromal cells. lncRNAs with FoldChange > 1.5 and false discovery rate (FDR) < 0.05 in the analysis were considered FibLnc. Based on this procedure, 95 lncRNAs were screened for subsequent analyses.

### FibLnc risk model construction

After identifying the differentially expressed genes, we further performed univariate Cox regression analysis on all lncRNAs based on clinical information from TCGA patients with breast cancer. For each lncRNA, we selected the median value of their expression as the cutoff, aliquoted the samples into two groups of high and low expression, and performed survival analysis. Finally, we screened all log-rank *p* < 0.05 lncRNAs as survival-related lncRNAs with CAF-specific expression in the breast cancer microenvironment and used them to construct prognostic models. The models were built based on the LASSO-Cox regression analysis function of the R package ‘glmnet’ v4.1-2, and seven lncRNAs were used to construct the final model after 1,000-time permutation and cross-validation. We defined the model risk score as the FibLnc score, and the FibLnc score was obtained by a linear combination of the expression of the seven genes and the corresponding regression coefficients, which could be represented as: FibLnc score = - 0.003 × HHLA3 - 0.002 × TP53TG1 – 0.519 × ST7-AS1 + 0.065 × LINC00536 + 0.304 × ZNF503-AS1 + 0.044 × MIR22HG – 0.392 × MAPT-AS1. We next divided TCGA breast cancer samples into high- and low-risk subgroups based on the optimal threshold for obtaining the maximum survival difference.

### Mutation analysis

Somatic mutation information for TCGA breast cancer samples was downloaded from Xena Browser (https://xenabrowser.net/datapages/). Due to the numerous mutation types, we did not make a distinction when analyzing the mutational landscape and differences. Based on previous studies, in counting the differences in TP53 in the two subgroups of high and low risk, mutations that do not affect protein expression were considered wild-type, whereas those that affect the entire protein sequence, such as coding frameshifts and nonsense mutations, were considered truncating mutations (41). The R package ‘maftools’ v2.6.05 was used to analyze the mutation differences between the high- and low-risk subgroups. Statistical significance was set at *P* < 0.05.

### Drug response analysis

We downloaded the gene expression matrix of 805 cell lines and their half-maximal inhibitory concentration (IC50) values under 198 drug treatments from the Genomics of Drug Sensitivity in Cancer database (42). Using the R package ‘oncoPredict’ v0.2, we used the downloaded data as a training set to build a ridge regression model, which was then applied to a new gene expression dataset to predict the clinical chemotherapy response. We predicted the IC50 values of the TCGA breast cancer samples for 198 drugs and normally transformed these values. We then used multiplicative changes in median values for the high- and low-risk subgroups to represent differences in drug response.

### Bioinformatics analysis

DEGs in the high-and low-risk subgroups were calculated using the R package ‘DESeq2’ v1.30.1. We selected the genes with |log2FoldChange| > 1 and FDR < 0.05, as DEGs. Based on the calculated fold change of each gene, we performed gene set enrichment analysis using the GSEA function of the R package ‘clusterProfiler’ v3.18.1 (43). The genes used for the enrichment analysis included both the tumor hallmark signaling pathway and the Kyoto Encyclopedia of Genes and Genomes (KEGG) signaling pathway. Survival analysis and curve plotting were performed using the R package ‘survminer’ v0.4.9. The time-dependent receiver operating characteristic (ROC) curve of TCGA patients with breast cancer with one-, three-, five-year survival was determined using the R package ‘timeROC’. The nomogram and calibration curves measuring the performance of FibLnc scores were visualized using the R package ‘RMS’ v6.2-0. The immune cell dysfunction score of TCGA samples was retrieved from the Tumor Immune Dysfunction and Exclusion database (http://tide.dfci.harvard.edu/download/). The IPS of TCGA breast cancer samples was downloaded from The Cancer Immunome Atlas (https://tcia.at/home). Generally, a higher tumor-infiltrating cell exclusive score and a lower IPS predict a worse response to immunotherapy.

## Data availability statement

The original contributions presented in the study are included in the article/**Supplementary Material**. Further inquiries can be directed to the corresponding author.

## Author contributions

ZL and ZHL designed and supervised the experiments. ZHL and JY performed the experiments and data analysis. ZHL, JY, and CL contributed to data analysis and predictor development. ZHL wrote the manuscript with contributions from all the authors. All authors contributed to the article and approved the submitted version.

## Acknowledgments

We would like to thank Editage (www.editage.com) for English language editing.

## Conflict of interest

The authors declare that the research was conducted in the absence of any commercial or financial relationships that could be construed as a potential conflict of interest.

## Publisher’s note

All claims expressed in this article are solely those of the authors and do not necessarily represent those of their affiliated organizations, or those of the publisher, the editors and the reviewers. Any product that may be evaluated in this article, or claim that may be made by its manufacturer, is not guaranteed or endorsed by the publisher.

## Supplementary material

Figure S1 Analysis of the differences in the distribution of immune and stromal scores in different **(A)** ages, **(B)** T, **(C)** N, **(D)** M and **(E)** TNM stages. **(F)** Survival analysis of the low and high immune scores.

Figure S2
**(A)** The partial likelihood deviance was calculated using multivariate Cox regression. **(B)** The regression coefficients were calculated using multivariate Cox regression.

Figure S3 Kaplan–Meier survival curves for patients in the GEO dataset assigned to high- and low-risk groups based on the risk score. **(A)** GSE1456 datasets. **(B)** GSE7390 datasets. **(C)** GSE16446 datasets. **(D)** GSE20685 datasets. **(E)** GSE20711 datasets. **(F)** GSE42568 datasets.

Figure S4
**(A)** Analysis of the differences in the distribution of MILnc scores in different ages, T, N and M stages. **(B)** Correlation between FibLnc score and immune/stromal score and tumor purity.

Figure S5 Nomogram analysis. **(A)** Nomogram composed of age, TNM stage, PAM50 subtypes and FibLnc score for the prediction of 1-, 3-, and 5-years OS probability. Calibration plot for the evaluation of the nomogram in predicting 1-year **(B)**, 3-years **(C)**, and 5-years **(D)** OS probability.

Figure S6 Mutation landscape difference between high- and low-risk subgroups.

Figure S7 Mutation co-occurrence and mutually exclusive patterns difference between high and low-risk subgroups.

Figure S8
**(A)** Correlation analysis of synonymous mutation counts and nonsynonymous mutation counts and FibLnc scores. **(B)** Correlation analysis of PD-1 and CTLA4 expression value and FibLnc scores. **(C)** Distribution of IPS score of patients under anti-CTLA-4 or anti-PD-1 treatment between high- and low-risk subgroups.

Table S1 Stromal and immune infiltration levels of TCGA BRCA samples calculated by ESTIMATE algorithm.

Table S2 Differentially expressed lncRNAs between fibroblasts and other cells.

Table S3 Univariate Cox regression analysis of survival time of the 75 CAF-specific lncRNAs.

Table S4 Differentially expressed genes between the low- and high-risk groups.

Table S5 Hallmark pathway enrichment of differentially expressed genes.

Table S6 KEGG pathway enrichment of differentially expressed genes.

Table S7 Inferred IC50 values of 198 drugs in patients with TCGA-BRCA gene expression profile.

## Abbreviations

TME, tumor microenvironment; lncRNAs, long noncoding RNAs; TCGA, The Cancer Genome Atlas; DEGs, Differentially expressed genes; IC50, half-maximal inhibitory concentration; KEGG, Kyoto Encyclopedia of Genes and Genomes; timeROC, time-dependent receiver operating characteristic curve; IPS, immunophenoscore.
