# Supplementary figures and images for: Cancer-associated fibroblasts-derived lncRNA signature as a putative biomarker in breast cancer
Source: Front Oncol. 2022 Nov 2;12:1028664. doi: 10.3389/fonc.2022.1028664 (PMC9667072; doi:10.3389/fonc.2022.1028664)

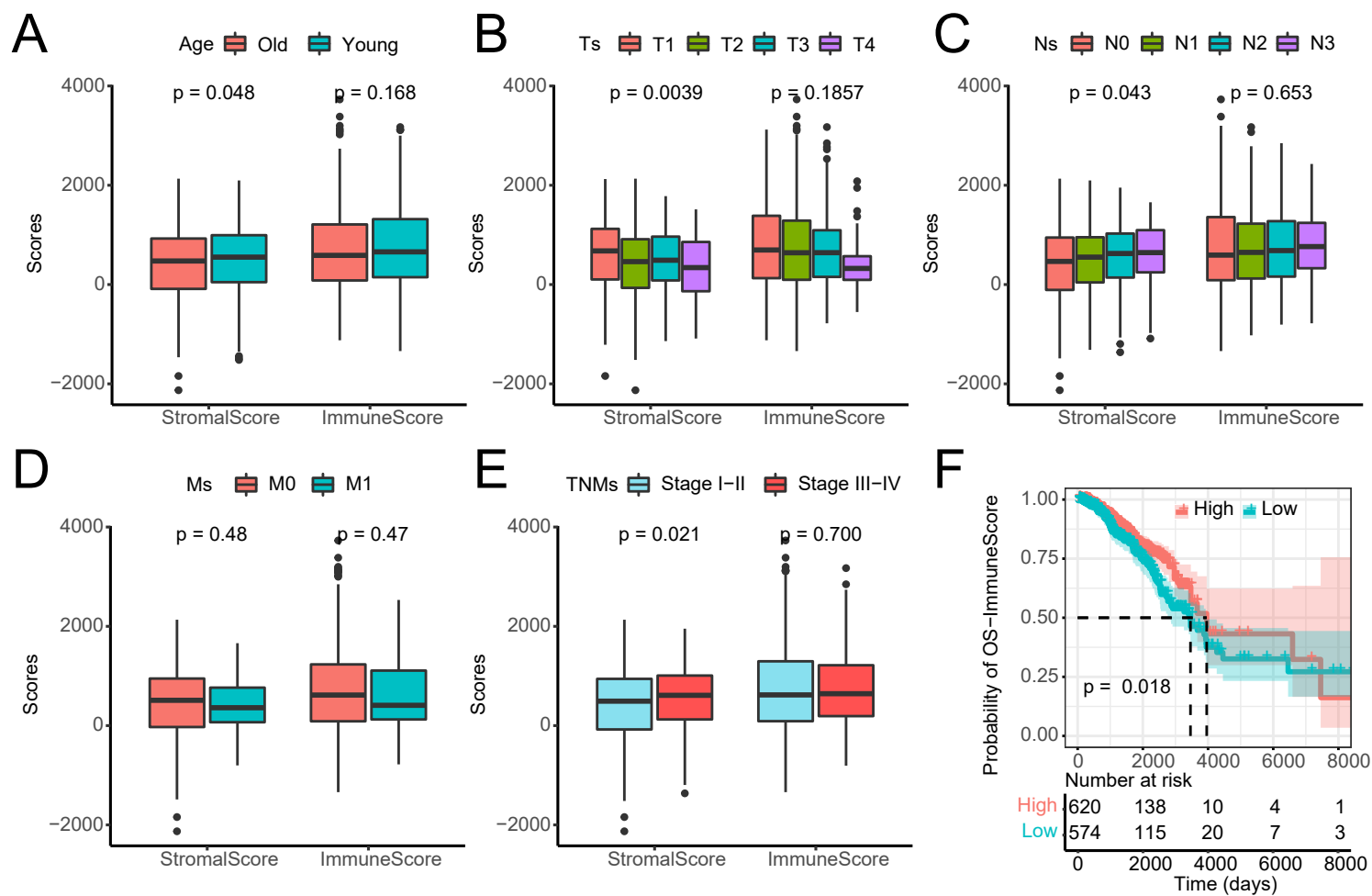

Supplement: Figure S1 — Analysis of the differences in the distribution of immune and stromal scores in different (A) ages, (B) T, (C) N, (D) M and (E) TNM stages. (F) Survival analysis of the low and high immune scores. [file Image_1.pdf]

**A**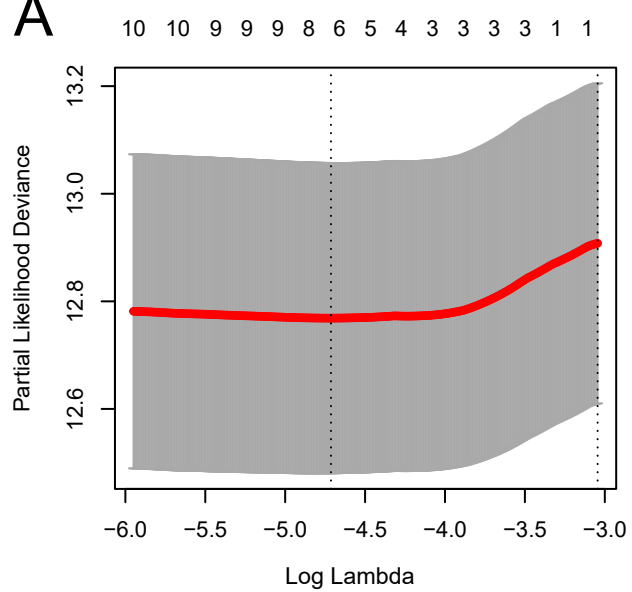**B**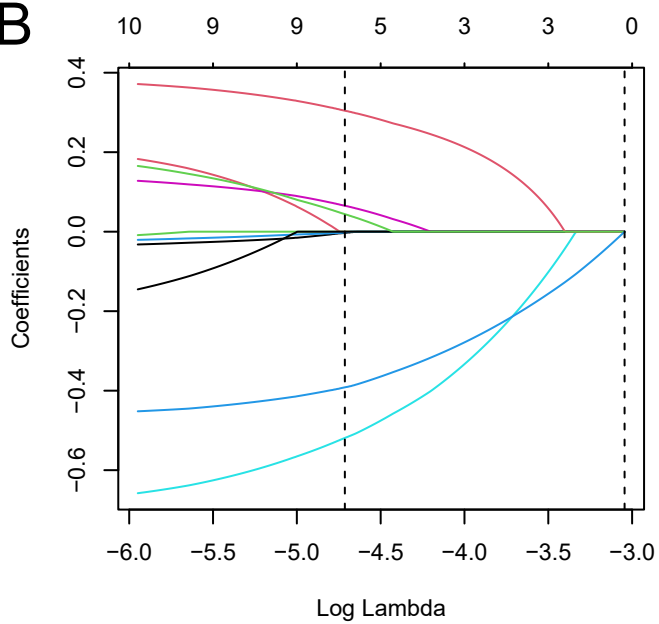

Supplement: Figure S2 — (A) The partial likelihood deviance was calculated using multivariate Cox regression. (B) The regression coefficients were calculated using multivariate Cox regression. [file Image_2.pdf]

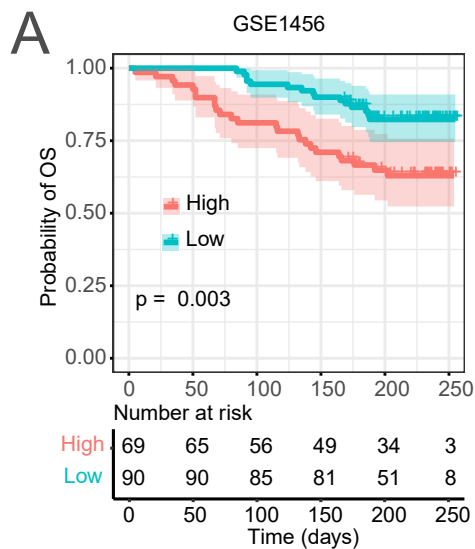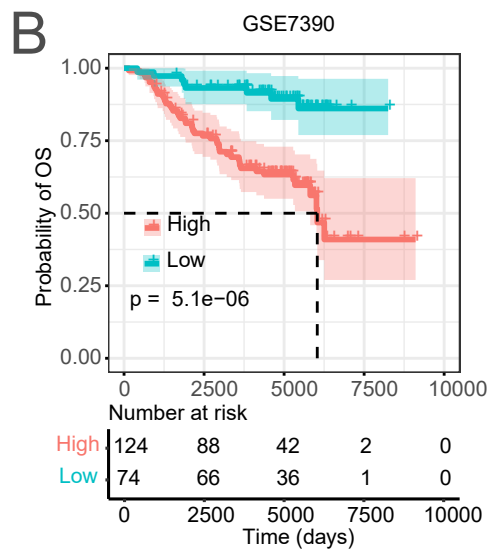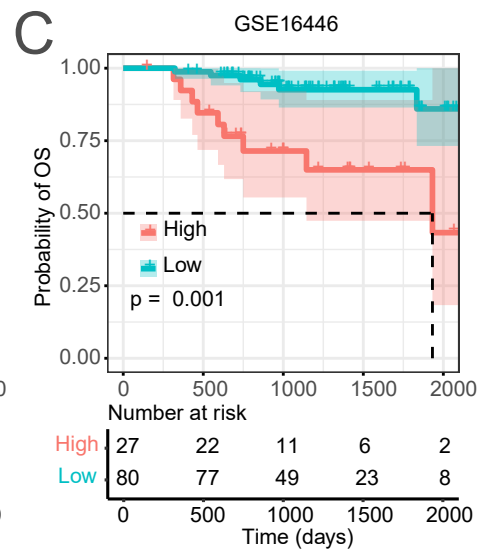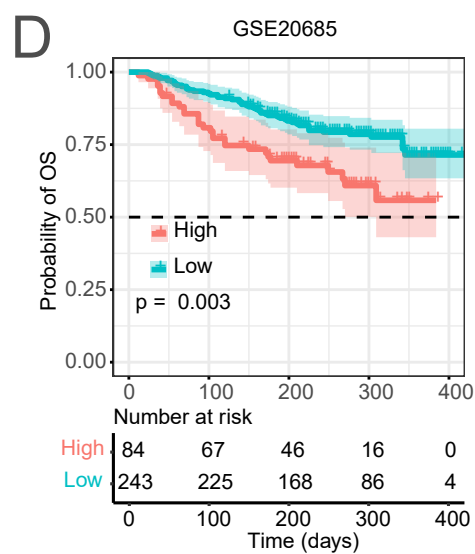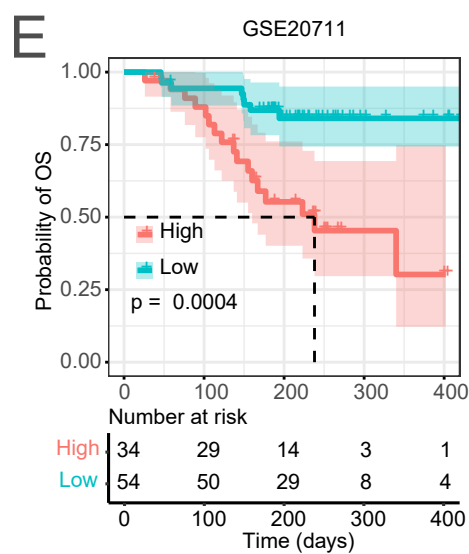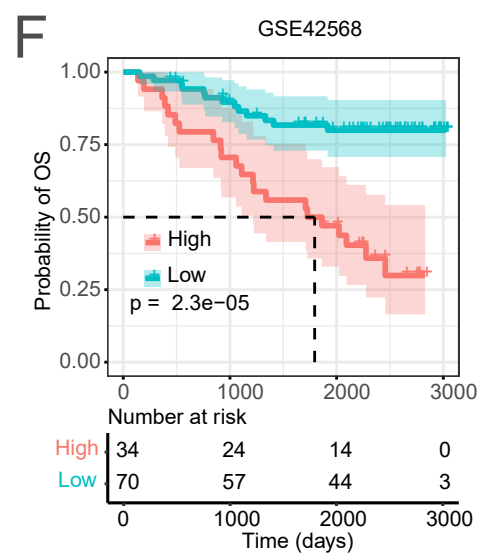

Supplement: Figure S3 — Kaplan–Meier survival curves for patients in the GEO dataset assigned to high- and low-risk groups based on the risk score. (A) GSE1456 datasets. (B) GSE7390 datasets. (C) GSE16446 datasets. (D) GSE20685 datasets. (E) GSE20711 datasets. (F) GSE42568 datasets. [file Image_3.pdf]

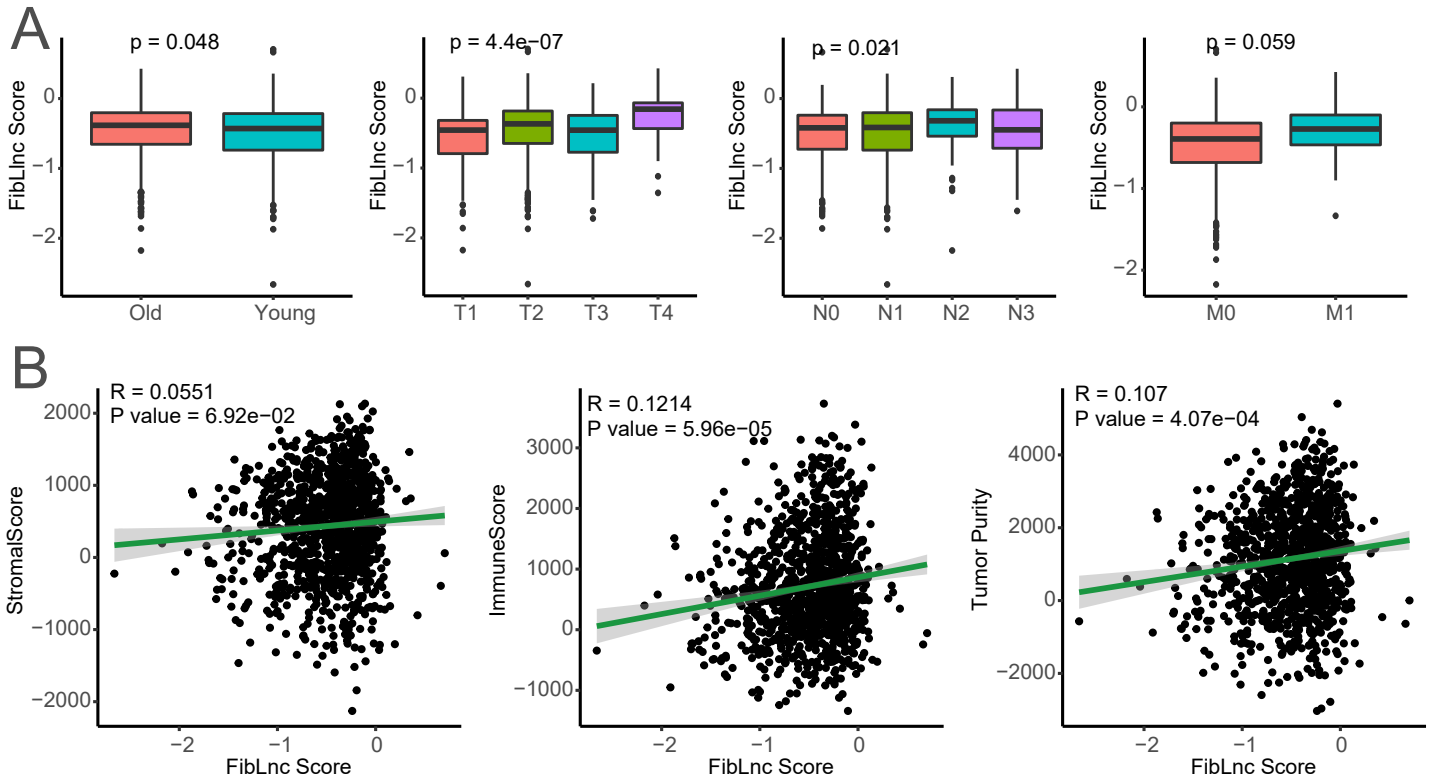

Supplement: Figure S4 — (A) Analysis of the differences in the distribution of MILnc scores in different ages, T, N and M stages. (B) Correlation between FibLnc score and immune/stromal score and tumor purity. [file Image_4.pdf]

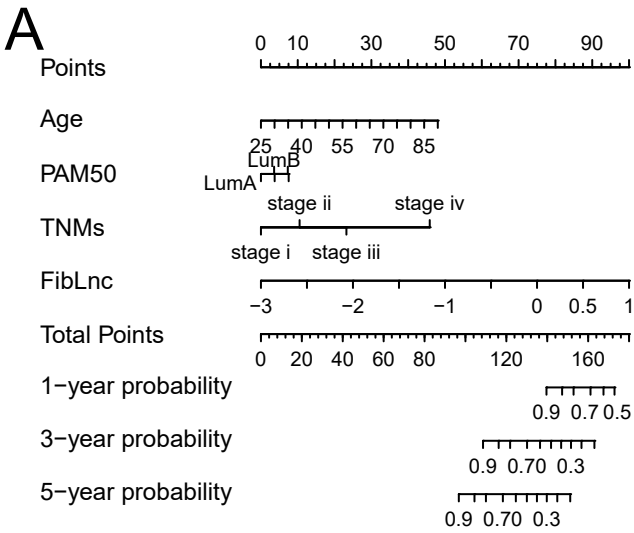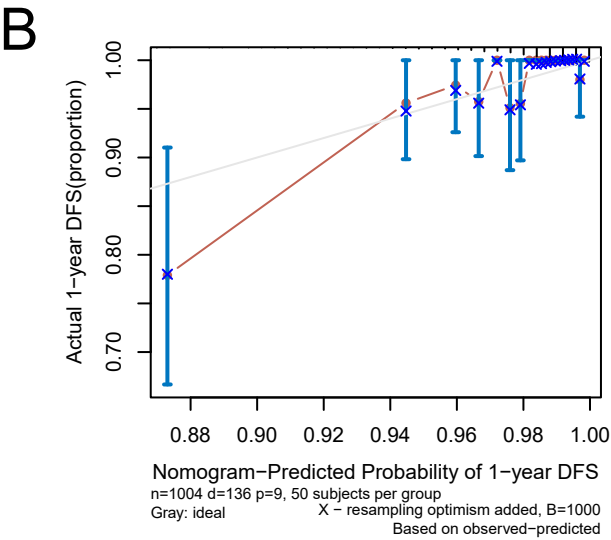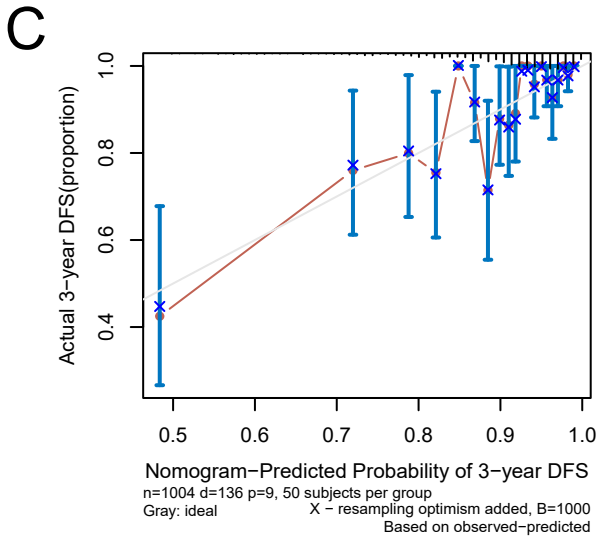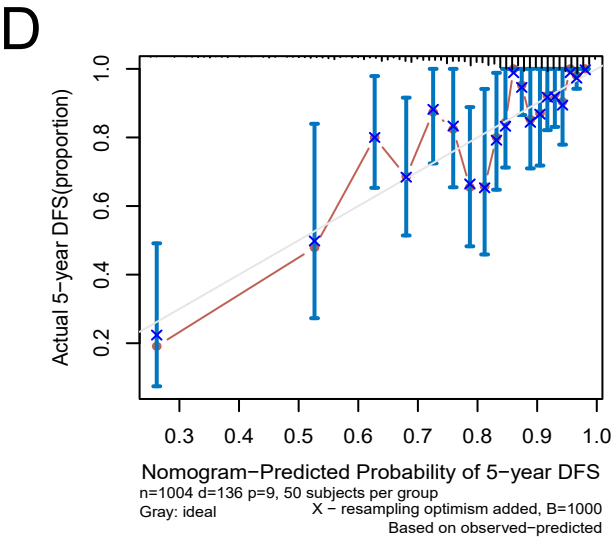

Supplement: Figure S5 — Nomogram analysis. (A) Nomogram composed of age, TNM stage, PAM50 subtypes and FibLnc score for the prediction of 1-, 3-, and 5-years OS probability. Calibration plot for the evaluation of the nomogram in predicting 1-year (B), 3-years (C), and 5-years (D) OS probability. [file Image_5.pdf]

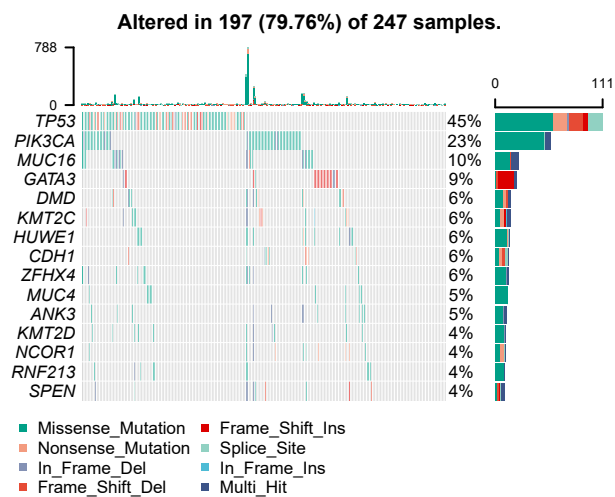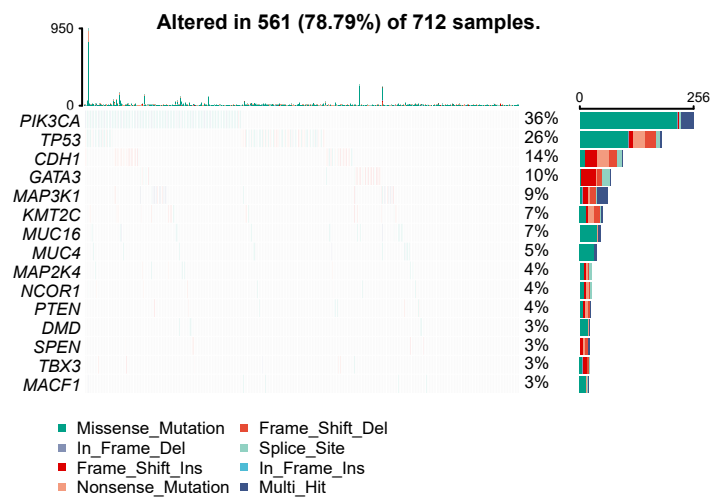

Supplement: Figure S6 — Mutation landscape difference between high- and low-risk subgroups. [file Image_6.pdf]

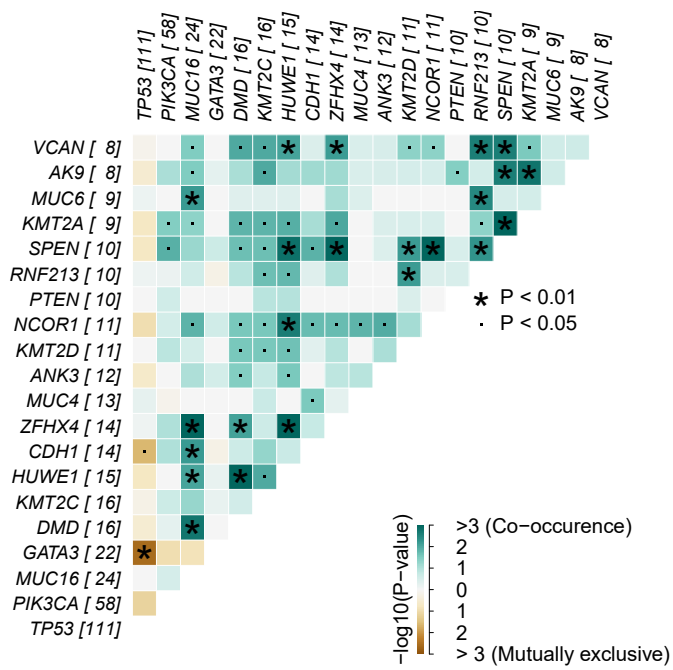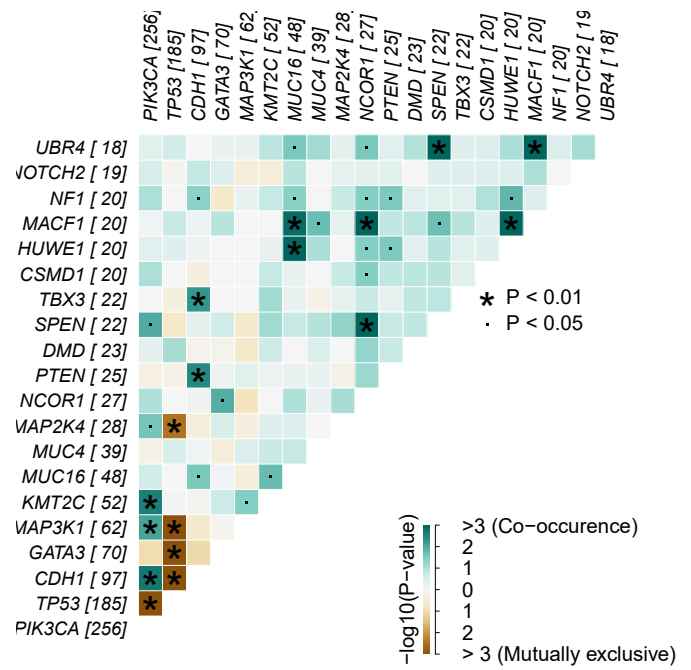

Supplement: Figure S7 — Mutation co-occurrence and mutually exclusive patterns difference between high and low-risk subgroups. [file Image_7.pdf]

A

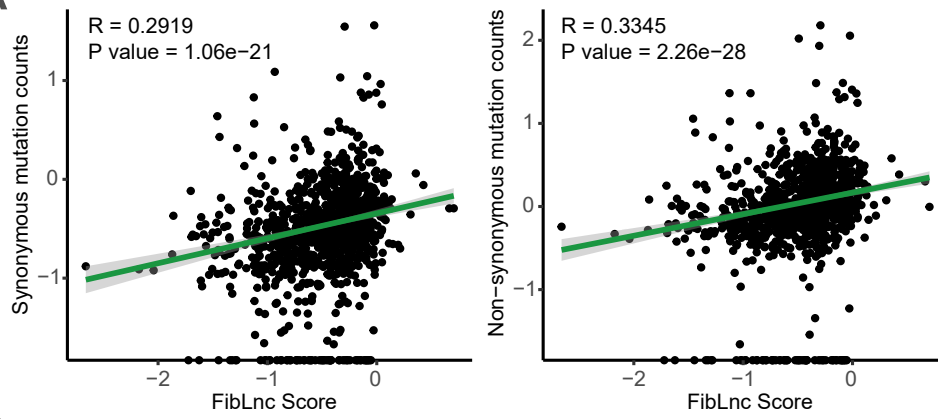

B

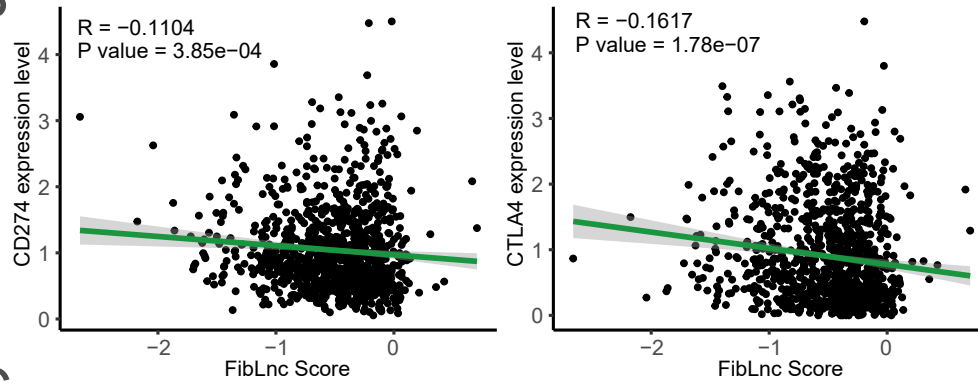

C

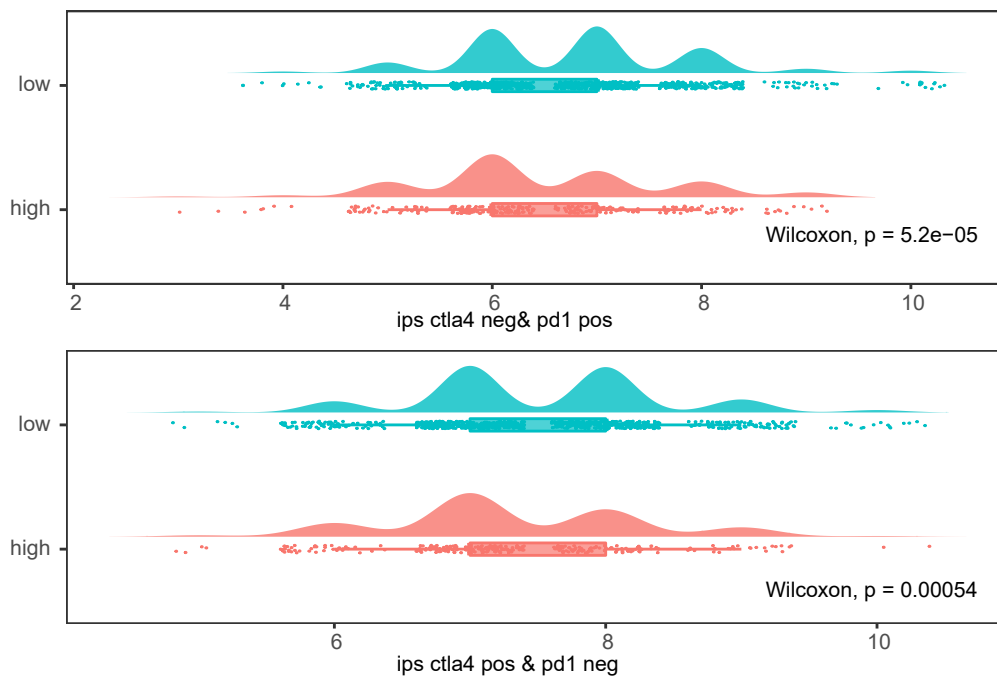

Supplement: Figure S8 — (A) Correlation analysis of synonymous mutation counts and nonsynonymous mutation counts and FibLnc scores. (B) Correlation analysis of PD-1 and CTLA4 expression value and FibLnc scores. (C) Distribution of IPS score of patients under anti-CTLA-4 or anti-PD-1 treatment between high- and low-risk subgroups. [file Image_8.pdf]
